# Harnessing digital health interventions to bridge the gap in prevention for older adults

**DOI:** 10.3389/fpubh.2023.1281923

**Published:** 2024-01-08

**Authors:** Kim Daniels, Bruno Bonnechère

**Affiliations:** ^1^Department of PXL – Healthcare, PXL University of Applied Sciences and Arts, Hasselt, Belgium; ^2^REVAL Rehabilitation Research Center, Faculty of Rehabilitation Center, Hasselt University, Diepenbeek, Belgium; ^3^Technology-Supported and Data-Driven Rehabilitation, Data Science Institute, Hasselt University, Diepenbeek, Belgium

**Keywords:** aging, digital health, prevention, happy aging, fall, sarcopenia, dementia

## 1 Introduction

The current global population of older adults is undergoing a notable and swift growth, which presents substantial health-related obstacles to public health systems on a global scale (1). The anticipated doubling of the aging population by 2050 has resulted in an increased incidence of age-related illnesses, including falls, sarcopenia, and dementia (2). The health issues associated with aging not only have a significant impact on the overall wellbeing of older individuals but also puts a lot of pressure of the healthcare system (3). The implementation of preventive measures aimed at addressing falls, sarcopenia, and dementia is therefore of utmost importance in order to minimize the negative impact of these conditions on the wellbeing, quality of life, and autonomy of older individuals (4, 5). Nevertheless, it is important to acknowledge that public health systems encounter distinct obstacles when it comes to efficiently tackling these concerns (6, 7). These constraints encompass for example restricted resources, insufficient infrastructure, and discrepancies in healthcare accessibility (1). The World Health Organization (WHO) highlighted five recommendations to promote physical activity: strengthen government (ownership and leadership), provide practical tools and guidance, support partnerships and build capacity, reinforce data systems and knowledge translation, secure and align funding with national policy (8). Therefore, it is crucial to prioritize the development and implementation of preventative measures that take into account the unique requirements and circumstances of older persons in various socioeconomic contexts. This is essential in order to promote healthy aging and enhance public health outcomes on a global scale (9).

## 2 The role of digital health interventions

In recent times, there has been a notable emergence of digital health treatments, such as wearables sensors, mobile health app, virtual reality, that show promise in the domain of preventative care for older adults (10). These interventions present creative solutions aimed at effectively addressing the health concerns faced by this demographic. The pervasive utilization of mobile health applications and wearable sensors has fundamentally transformed the delivery of healthcare, enabling the provision of real-time monitoring, tailored interventions, and remote healthcare assistance (11). The use of digital platforms has a unique and unparalleled prospect to effectively engage a broader demographic of older individuals, while simultaneously reducing expenses in comparison to conventional healthcare approaches (12). Through the use of technological advancements, digital health interventions have the potential to enable older adults to actively engage in the management of their health, hence facilitating early identification, prevention, and the provision of individualized care. The potential for altering public health methods and improving healthcare solutions for older individuals globally is considerable through the seamless integration of these technologies into preventive initiatives (13, 14).

## 3 Addressing physical function

Falls and sarcopenia are prominent health issues that exert a substantial influence on the overall welfare and autonomy of older adult individuals. The older population is particularly vulnerable to falls (15–17), which present a substantial risk resulting in injuries, hospitalizations, and reduced mobility (18, 19). Sarcopenia, an age-related phenomenon characterized by the progressive reduction in muscle mass and strength, is a significant factor in the development of frailty and functional decline among older adults (20). This condition increases the risk of falls and several other health concerns.

In order to tackle these aforementioned issues, the utilization of wearable sensors and digital health has emerged as a valuable means for assessing fall risk and providing balance training in the older population (21, 22). The sensors have the ability to track alterations in gait and balance, hence facilitating the prompt recognition of persons who may be susceptible to falling. Wearable sensors facilitate the customization of balance training programs for individuals by offering real-time data on gait patterns and postural stability (23). This capability empowers healthcare providers to mitigate the likelihood of falls and enhance overall mobility.

In conjunction with fall prevention, mobile applications present considerable opportunities for strength training and tele-rehabilitation among the older population (24). These applications have the ability to provide personalized workout routines that specifically focus on particular muscle groups, hence assisting in the reduction of sarcopenia's impact and enhancing overall physical functionality (25). In addition, the use of mobile applications for telerehabilitation facilitates the provision of rehabilitation services from a distance, therefore offering significant advantages to older adult individuals residing in geographically isolated or underserved regions. This technique facilitates self-regulation and compliance with exercise protocols, hence encouraging sustained enhancements in functionality and autonomy among older adults.

Through the use of wearable sensors and mobile applications, healthcare practitioners have the ability to customize preventive treatments (i.e., personalized rehabilitation) to the exact needs and abilities of the older adults, thereby effectively addressing issues such as falls, sarcopenia, and other health-related concerns. Digital health solutions have the capacity to increase the quality of life for older individuals, diminish healthcare expenditures, and foster the promotion of healthy aging inside a progressively aging global population (26).

## 4 Enhancing cognitive health

The increasing prevalence of dementia and cognitive impairment in older populations has emerged as a major public health concern (27). The increasing worldwide population aging phenomenon has led to a corresponding rise in the prevalence of cognitive illnesses, such as dementia. These disorders not only have an impact on an individual's cognitive ability but also have substantial effects on their daily functioning and overall quality of life. Additionally, they impose a burden on healthcare systems and carers (28, 29).

To overcome this challenge, the use of digital tools has emerged as a valuable means of cognitive assessment and monitoring among older persons (30). These instruments have the capability to offer impartial and consistent assessments of cognitive abilities, hence enabling the timely identification and implementation of appropriate measures. Through the use of digital cognitive evaluations, healthcare providers are able to discern minor alterations in cognitive capabilities (31). This enables them to implement interventions that are both timely and individualized, with the aim of mitigating or managing cognitive decline.

In addition, cognitive training applications present encouraging prospects for enhancing cognitive abilities among the older population (32). These applications often comprise of stimulating and interactive activities that are specifically developed to address several cognitive areas, including memory, attention, and problem-solving. The consistent utilization of these applications has demonstrated promise in augmenting cognitive functioning and fostering neuroplasticity among older adults (33). The customization of cognitive training allows for an individualized and inclusive method to effectively target cognitive decline in older people.

The incorporation of digital tools in cognitive healthcare presents a promising prospect for revolutionizing our approach to addressing dementia and cognitive decline in the older population. The use of digital assessments and cognitive training applications possesses the capacity to facilitate timely intervention and boost cognitive wellbeing in older populations. The use of digital therapies becomes progressively imperative in addressing the escalating incidence of cognitive disorders and promoting healthy aging among the aging global population (34).

## 5 Challenges and solutions

The implementation of digital health treatments for older individuals is hindered by the considerable hurdles posed by the insufficiency of resources and the inadequacy of healthcare infrastructure. The lack of sufficient technology, internet connectivity, limited digital literacy skills, data privacy, security threats, limited device performance, and healthcare infrastructure can impede the widespread implementation of digital solutions in these particular places (35). Furthermore, the limited availability of adequately qualified healthcare personnel and financial limitations may pose obstacles to the advancement and execution of digital health initiatives targeting the older population (36).

In order to optimize the efficacy of digital treatments, it is imperative to customize and designing these solutions with older adults to align with the cultural and educational contexts of older individuals across various geographical areas (37). The design and execution of digital health initiatives necessitate the consideration of cultural views, linguistic preferences, and diverse degrees of digital competence. By tailoring interventions to be congruent with local cultures and educational levels and gaining insight into the technical challenges that older adults encounter will empower developers to craft future digital interventions that are precisely attuned to their needs (38).

The successful implementation of digital health interventions necessitates the resolution of obstacles pertaining to the acceptability and utilization of technology among the senior population. Older adult individuals may exhibit apprehension about technology due to perceived complexities and apprehensions around privacy and data security (39). Addressing these concerns is of utmost importance by implementing user-friendly interfaces, fewer buttons, larger text, improved color contrast, providing clear instructions, and adopting transparent data management methods (40). The present systems struggle to cope with the demands of processing and securing the vast influx of multi-sensory data captured. Employing advanced data processing techniques becomes imperative to seamlessly integrate the rich of information acquired from wearable sensors, translating it into clinically relevant outputs. The provision of sufficient training and assistance for older persons in the utilization of digital technologies has the potential to enhance their self-assurance and self-efficacy and ease in engaging with these treatments for the purpose of promoting their health and overall wellbeing. In addition, implementing rigorous regulations, fostering strong societal support, and engaging proactively with older adults can contribute to enhancing their utilization and confidence in digital healthcare. This multifaceted approach also ensures proper usage, privacy, and security. Digital health treatments have the potential to significantly narrow the healthcare disparity among older individuals in low- and middle-income countries by effectively acknowledging and tackling the obstacles related to limited resources, cultural diversity, and adoption of technology (41). Tailored digital solutions possess the capacity to empower the older population, enhance their accessibility to preventive healthcare, and augment their general wellbeing and quality of life. Furthermore, through the promotion of cooperation among policymakers, healthcare practitioners, and technology innovators, it is possible to collectively establish enduring and all-encompassing digital health initiatives that yield advantages for older individuals in many socioeconomic contexts (42).

## 6 Future directions

**Figure 1:**
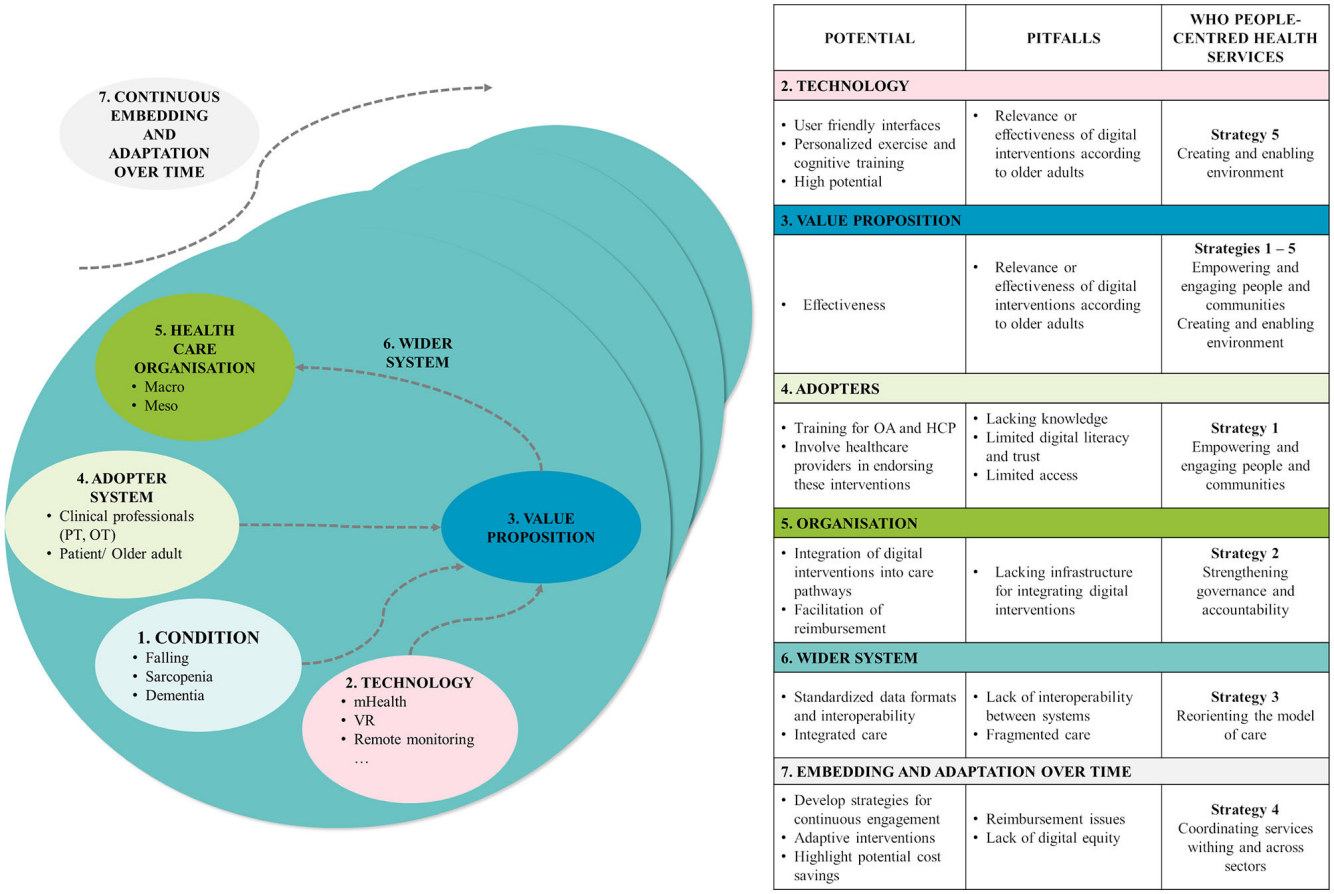
NASSS framework (43) adapted for the adoption of digital technologies in aging using the WHO people centered health services (44).

Table 1 displays our proposed suggestions for advancing research and enhancing the efficacy of digital health interventions targeted toward the older population. The table was divided into three distinct areas, including the suggestions, the possible impact of digital health on the healthcare sector, and initiatives aimed at ensuring sustainability. In Figure 1, we presented an adapted version of the NASSS framework (Non-adoption, Abandonment, Scale-up, Spread, Sustainability) as an analytical tool to depict both the challenges and opportunities (43). This framework offers a comprehensive perspective on the complexities associated with implementing healthcare technologies, particularly in the context of older adults. The NASSS framework acknowledges that the successful adoption of technology depends on a myriad of factors, encompassing technological, organizational, social, and contextual dimensions. Given the unique challenges inherent in introducing digital health interventions to older adults, the NASSS framework proves invaluable in identifying potential obstacles and essential considerations throughout the implementation process. Through the application of this framework, we delved into various facets, including the interaction between technology and the older adult population, organizational readiness, societal attitudes, and the overall ecological context.

**Table 1:** Recommendations, potential and strategies to ease the implementation of digital health for prevention in older adults.

**1. Recommendations for further research and development**	**2. Potential of digital health in integrating with healthcare systems**	**3. Strategies for ensuring long-term efficacy and sustainability**
**1.1 Conduct longitudinal studies** In order to ascertain the enduring efficacy of digital health interventions, it is imperative for researchers to do longitudinal studies that systematically monitor the influence of these interventions on the health outcomes of older persons over a protracted duration. Longitudinal data possesses the capacity to yield significant insights pertaining to the enduring advantages of digital interventions, as well as facilitate the identification of elements that contribute to favorable long-term outcomes	**2.1 Collaborate with healthcare providers** Promote synergistic engagement between innovators in the digital health field and healthcare professionals, with the aim of effectively integrating digital interventions into established healthcare systems. This collaborative effort has the potential to facilitate the sharing of data, enhance the coordination of care, and ultimately improve the entire healthcare experience for the older population	**3.1 User engagement and empowerment** The primary focus should be on enhancing user involvement and empowerment by implementing user-friendly interfaces, providing comprehensive training, and offering continuous support. There is a higher likelihood of sustained usage of digital health treatments and adherence to suggested health practices among users who are actively engaged and empowered
**1.2 Personalized interventions** The primary objective is to prioritize the advancement of tailored digital health solutions that effectively address the specific requirements, inclinations, and medical circumstances of older adult individuals. The utilization of artificial intelligence and machine learning algorithms has the potential to enhance the customization of interventions and adjust them to the changing needs of older persons over time	**2.2 Telemedicine and remote monitoring** This study aims to investigate the possibilities of telemedicine and remote monitoring technologies in expanding healthcare services to underserved populations residing in remote or rural locations. Digital health interventions have the potential to enhance healthcare accessibility for older persons who face reduced mobility or reside in areas with limited healthcare services. This can be achieved through the facilitation of virtual consultations and remote health monitoring, effectively overcoming geographical obstacles	**3.2 Continuous improvement and updates** It is imperative to consistently enhance and refine digital health solutions by incorporating user feedback and integrating the most current scientific evidence. The utilization of iterative development methodologies has the potential to improve the efficacy and pertinence of interventions, hence resulting in enhanced health outcomes for the older population
**1.3 Evaluate cost-effectiveness** Evaluate the cost-effectiveness of digital health initiatives in contrast to conventional healthcare methodologies. Comprehending the economic ramifications will be of utmost importance for policymakers and healthcare providers when assessing the viability of widespread implementation of digital health solutions	**2.3 Public-private partnerships** The establishment of public-private partnerships is recommended to effectively harness the respective strengths of both the public and private sectors in the development and implementation of digital health solutions. Public-private collaborations have the potential to enhance the implementation of digital health interventions on a wider scope and promote inclusivity for marginalized communities	**3.3 Scalability and adaptability** When designing digital health interventions, it is imperative to consider scalability and adaptability as key factors. The solutions ought to possess the capacity for seamless expansion in order to cater to a broader demographic and be flexible enough to accommodate a wide range of cultural, educational, and linguistic contexts
**1.4 Establish collaborative research networks** To enhance research skills, methodologies, and overall capacity on a global scale, it is imperative to allocate resources and efforts across high, middle, and low-income countries. This approach recognizes the significance of promoting equitable development and knowledge-sharing across diverse socioeconomic contexts. By focusing on these key areas, we can drive advancements in various fields and foster collaboration among researchers worldwide	**2.4 Health apps and wearable devices** The integration of health apps and wearable devices into healthcare systems allows for real-time health data collection, tracking, and analysis. These tools can assist in preventive care, disease management, and offering personalized health recommendations	**3.4 Prioritize staffing and funding** Carry out partnerships is essential for the success of collaborative efforts. Partnerships in various sectors, whether they involve businesses, non-profit organizations, government agencies, or academia, can bring diverse expertise and resources together to tackle complex challenges
**1.5 Prioritize dissemination and implementation research** Bridging the gap between research findings and practical application is a complex challenge that requires a strategic approach to ensure that valuable scientific insights translate into real-world benefits	**2.5 Global health initiatives** Digital health has the potential to play a transformative role in bridging healthcare gaps in underserved areas by leveraging technology to provide essential medical services and information (i.e., health literacy)	**3.5 Behavior changes theories** Integrating interventions into digital health solutions is key to effectively encouraging and sustaining healthy behaviors. Digital platforms provide unique opportunities to deliver personalized, engaging, and timely interventions that can drive positive behavior change

Furthermore, we integrated the NASSS framework with the WHO People-Centered Health Service Strategies. These strategies underscore the significance of tailoring health services to individual needs, preferences, and experiences. By combining these frameworks, we offer a more holistic understanding of the interplay between technology implementation and the human-centric aspects of healthcare delivery for older adults. This synergistic approach allows us to explore not only the technical intricacies of digital health interventions but also their alignment with the preferences and values of the older adult population.

## 7 Conclusion

The urgent need for quick action is evident in the imperative to address the preventative gap among older persons. The prevalence of age-related illnesses, including falls, sarcopenia, and dementia, is increasing in tandem with the global aging population. In the absence of efficient preventive measures, the welfare and autonomy of older adult individuals are in jeopardy, potentially leading to significant burdens on healthcare systems in terms of delivering sufficient care and assistance. The prompt emphasizes the importance of promptly and resolutely implementing certain preventive measures aimed at mitigating the effects of these disorders on the lives of older adults.

The incorporation of digital health interventions presents a potentially effective approach to improve the physical function and cognitive health of older adult individuals. Through the use of wearable sensors, smartphone applications, and tele-rehabilitation platforms, digital treatments have the potential to enable older adults to actively engage in the management of their health. These interventions have the potential to enhance the timely identification of health hazards, provide tailored exercise and cognitive training programs, and encourage the adoption of healthy behaviors. In addition, the accessibility and scalability of digital health solutions have the potential to expand their impact on older persons residing in distant or underserved regions, hence mitigating gaps in healthcare accessibility.

The transformative impact of digital health interventions on preventative care for older individuals should not be underestimated. By providing older adults with the tools and resources to actively monitor their health, participate in preventive activities, and effectively manage chronic illnesses, these interventions possess the potential to significantly improve overall wellbeing and foster healthy aging on a global level. The digital health revolution has far-reaching effects that transcend beyond the realm of individual health outcomes. It has the potential to alleviate the strain on healthcare systems, mitigate healthcare expenditures, and enhance the overall quality of life for older persons and their careers.

In summary, it is of utmost importance to address the pressing need for closing the gap in preventative measures for the older population. The incorporation of digital health interventions into the realm of preventative care for older individuals on a global scale presents a remarkable prospect for advancing physical function and cognitive health. This has the potential to bring about a transformative shift in the field. By strategically allocating resources toward research, development, and implementation endeavors, it is possible to enhance the autonomy and wellbeing of older adults, thereby securing a more promising outlook for aging populations on a global scale.

## Author contributions

KD: Visualization, Writing—original draft, Writing—review & editing. BB: Conceptualization, Supervision, Writing—original draft, Writing—review & editing.
